# Association of pigment epithelium derived factor expression with cancer progression and prognosis: a meta-analysis study

**DOI:** 10.1007/s12672-021-00457-y

**Published:** 2021-12-15

**Authors:** Guo Cheng, Crystal Song

**Affiliations:** Department of Physiology, Stein Eye Institute, David Geffen School of Medicine, Jonsson Comprehensive Cancer Center, University of California, Los Angeles, CA USA

**Keywords:** Pigment epithelium derived factor (PEDF), Cancer progression, Meta-analysis, Overall survival, Prognosis

## Abstract

**Background:**

Pigment epithelium derived factor (PEDF) is a secreted protein that strongly suppresses angiogenesis and directly inhibits cancer cells proliferation. The differential expression of PEDF has been observed in multiple types of human tumors. However, it is unclear as to how PEDF expression is associated with cancer progression and if PEDF could serve as a prognostic marker for cancer patients.

**Methods:**

We performed a comprehensive search for the studies on PEDF expression in 14 top-ranked types of solid tumor cancer with the highest incidence. A systemic approach was used to screen for qualified studies and to extract data. Meta-analysis was performed to investigate if PEDF expression is associated with the TNM staging, tumor size, lymph node invasion, distal metastasis and pathological grade of tumor in a pan-cancer manner. A Kaplan–Meier curve was plotted with the digitally-reconstituted patient survival data to study the effect of PEDF expression on the prognosis of cancer patients.

**Results:**

A total of nine studies were selected, reviewed and analyzed. Meta-analysis suggested that decreased PEDF protein expression was associated with higher TNM staging (OR = 2.13, 95% CI: 1.61–2.81), larger tumor size (OR = 1.42, 95% CI: 1.1–1.84), larger possibility of lymph node invasion (OR = 1.68, 95% CI: 1.26–2.22) and higher pathological grade (OR = 1.6, 95% CI: 1.2–2.13). No correlation was found between PEDF expression and tumor distal metastasis, gender or age. In addition, low PEDF protein level in tumor tissue is correlated with shorter overall survival (P < 0.05).

**Conclusions:**

Low PEDF protein expression in cancer is significantly associated with more advanced cancer progression and significantly poorer survival. The differential clinical outcome among patients with various PEDF expression suggests its prognostic value.

**Supplementary Information:**

The online version contains supplementary material available at 10.1007/s12672-021-00457-y.

## Introduction

Pigment epithelial-derived factor (PEDF) was first identified as a protein factor secreted by human retinal pigment epithelium cells [1]. It is also called serine protease inhibitor F1 (SERPINF1) and belongs to the serine protease inhibitor (serpin) superfamily. However, it has no serine protease inhibitor activity [2]. Numerous previous studies suggested that PEDF is a protein with multifaceted anti-tumor activities. Its most recognized function is the ability to inhibit angiogenesis, a process that drives tumor growth and metastasis [3, 4]. In in vitro assays, PEDF’s anti-angiogenic activity was found to be more potent than other endogenous anti-angiogenic factors such as endostatin, angiostatin and thrombospondin-1 [5]. In a variety of in vivo models, PEDF reduced microvascular density in tumor tissues which is one of the mechanisms leading to tumor inhibition [6–11]. PEDF’s receptor that mediates its antiangiogenic activity has been identified as a cell-surface transmembrane protein Plexin domain-containing protein 1 (PLXDC1) which is also known as tumor endothelial marker 7 (TEM7) [12]. PLXDC1 and its homologue PLXDC2 are the only two proteins that have been demonstrated to confer cell surface binding to extracellular PEDF and to transduce PEDF signal into the cell. PLXDC1/TEM7 was highly enriched in the blood vessels of tumor tissues but not in the blood vessels of adjacent normal tissue. PLXDC1’s tumor blood vessel expression was found in a wide range of cancer types, including liver cancer, breast cancer, ovarian cancer, pancreatic cancer, colorectal cancer, lung cancer, neuroblastoma and sarcomas [13–17]. This high specificity of PEDF receptor’s expression in tumor blood vessels matches the specificity of PEDF’s tumor inhibitory effect.

Other than inhibiting angiogenesis, PEDF also directly inhibits certain tumor cells and promotes cell differentiation [18]. Previous studies showed that PEDF can suppress the growth of lung cancer cells [19] and promote apoptotic cell death in melanoma cells [20, 21]. In addition, PEDF was found to induce the differentiation of tumor cells, such as eliciting neuron-like morphology in neuroblastoma cells [22] and promoting neuroendocrine function of prostate cancer cells [23]. Interestingly, another PEDF receptor, PLXDC2 was found to be expressed in a variety of cancers, including colon cancer, hepatocellular carcinoma, laryngeal cancer, testicular seminoma, and vulva squamous cell carcinoma [24–28]. PLXDC2-mediated signaling could be responsible for the direct effect of PEDF on cancer cells. The collective anti-cancer activity (anti-angiogenesis and direct anti-tumor cell effect) of PEDF was observed in in vivo studies. PEDF treatment by gene therapy or administration of recombinant protein was shown to inhibit the growth of pancreatic cancer [8], hepatoblastoma [6], prostate cancer [7], retinoblastoma [10], ocular melanoma [11], lung cancer and colon cancer [9]. It also significantly reduced thoracic metastasis of colon cancer [9].

PEDF is widely expressed in most human organs and tissues such as eye, liver, heart, brain, bone and lung [29]. A significant decrease of PEDF level was found in age-related macular degeneration (AMD) and diabetic retinopathy (DR), two pathological processes dependent on angiogenesis. PEDF expression in cancer was also studied in a wide range of cancer types. Although the association between PEDF expression and tumor development has been reported by many studies, each result only applies to individual cancer and there is no broad view on PEDF’s effect on diverse types of cancer. Given the multifaceted anti-tumor effects of PEDF and the presence of PEDF signaling machinery in various types of cancer, it would be interesting to know if PEDF protein expression level is associated with cancer progression and the prognosis of patients in general. To answer this question, we use systemic review and meta-analysis to inclusively identify research on this topic, unbiasedly select qualified studies and reach a weighted average with pooled data from the selected studies. Specifically, we reviewed the studies on PEDF expression in 14 types of top-ranked solid tumor malignancies with the highest incidence worldwide. We investigated if PEDF protein expression in cancer tissue is associated with the clinical development (TNM staging) and pathological feature (cancer grade) of tumors in a pan-cancer pattern by meta-analysis. We also explored the prognostic value of PEDF protein and discussed its biological importance and other potential values in cancer management.

## Method

### Search strategy

PubMed and EMBASE were searched for studies on PEDF expression in cancer through December 2020. The search included the 15 most prevalent cancer types worldwide in 2020 (the 13th-ranked cancer is leukemia and thus 14 types of solid tumor cancers were included in the study) [30]. No language restrictions were imposed. The search strategy was built using the key words for PEDF and the key words for each type of cancer. The 14 malignancies and the search strategies are summarized in Table S1.

### Study selection

Studies were included for meta-analysis if they meet the following criteria: (1) had a case–control design; (2) reported PEDF expression levels in human samples; (3) presented categorical data as case number. The search results and the literature were reviewed by two authors (G.C. and C.S.) independently.

### Data extraction and quality assessment

Data extraction form was designed to record data from selected studies by two reviewers (G.C. and C.S.) independently. The following information was extracted: the first author’s last name, year of publication, study design, age, gender, total number of cases, PEDF detection method, number of cases categorized based on age, gender, tumor TNM stage, tumor size, lymph-node invasion, distal metastasis, and tumor histopathological grade. Quality assessment was conducted using Newcastle–Ottawa Scale (NOS) and studies with an NOS score ≥ 7 were considered high quality [31].

### Reconstructing individual patient data of survival

Individual patient data (IPD) of survival were reconstructed from the published Kaplan–Meier curve (KMC) by the following steps: (1) KMC of overall survival (OS) was copied from the original study by Microsoft Windows “snipping” tool. (2) Published KMC was uploaded to the online data extraction tool “WebPlotDigitizer” [32] to extract survival probability (S_ti_) and time point (t_i_) from the curve. Data were extracted at the time point of every 10 months. (3) Due to the missing number at risk (N_ti_) in the published studies, we did approximate calculation of N_ti_ with the formula “N_ti_ = (S_ti-10_/S_ti_) × N_ti-10_-C_ti_”. The number of censored patients (C_ti_) were simplified as zero as five out of seven studies did not reveal this number. (4) S_ti_, t_i_ and N_ti_ were input to the algorithm in R developed by Guyot et al. [33] for the generation of IPD. IPD from all 7 studies were pooled for pan-cancer survival analysis.

### Retrieving individual patient data of survival from The Cancer Genome Atlas (TCGA)

We used an online platform, Oncolnc, which incorporates TCGA database and provides users with the access to IPD on gene expression (RNA level) and survivorship for 21 common malignancies [34]. IPD were sorted into two groups on Oncolnc: low gene expression (lower 50 percentile mRNA level) and high gene expression (higher 50 percentile mRNA level). IPD of 21 malignancies which composed of PEDF mRNA level and survival information of 7970 cases were downloaded and pooled for Kaplan–Meier curve plotting.

### Statistical analysis

Meta-analysis was performed if PEDF expression and clinical feature were reported in more than two studies. Pooled odds ratio (OR) and 95% confidence intervals were calculated to estimate the strength and credibility of the association. Either fixed-effect (*I*^*2*^ ≤ 50%, P ≥ 0.05) or random-effect (*I*^*2*^ ≥ 50%, P < 0.05) model was chosen based on the heterogeneity test [35]. The *I*^2^ test was used to assess the heterogeneity among studies. The *I*^*2*^ value was explained as of no (0–25%), low (25–50%), moderate (50–75%) or high heterogeneity (75–100%) [36]. Publication bias was evaluated with funnel plots. If publication bias was detected, it would be corrected by trim-and-fill method. Sensitivity test was performed by omitting one study at a time to assess the robustness of the pooled results (leaving-one-out method). Statistical analysis were conducted using the software Rstudio (version 1.3.1093). Package “meta” and “ggplot” were used for data analysis and graph generation.

Kaplan–Meier curve was created with “survfit” and “ggsurvplot” in Rstudio (version 1.3.1093). Pooled, reconstructed IPD were imported and the curve was plotted with a 10-month interval for 100 months. IPD downloaded from Oncolnc were pooled and plotted into Kaplan–Meier curve with a 20-month interval for 360 months.

## Result

### Systematic review and summary of selected studies

**Fig. 1:**
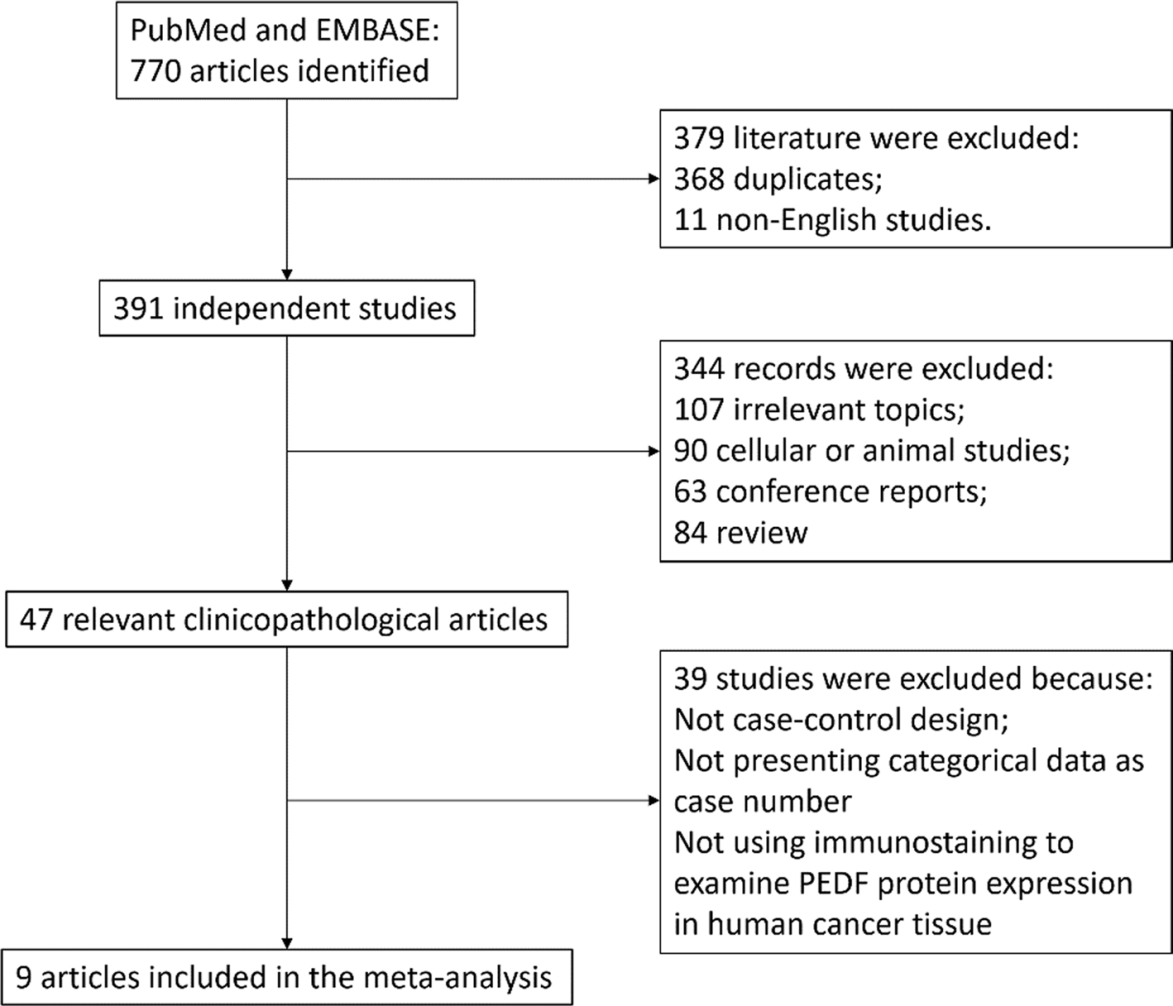
Flow diagram and results of literature review. The flow diagram depicts the step-wise screening of the retrieved articles from database, including the number and reasons of exclusion. A total of 9 studies were selected by this unbiased process

The search of PEDF expression in cancer generated a total of 770 articles. After removing those that are duplicated or not published in English, 391 literature remained. We further excluded 107 articles with irrelevant topics, 90 studies using *in* *vitro* or animal models, 63 conference reports and 84 review articles. Forty-seven articles are left that reported PEDF expression in human samples acquired clinically. Among them, 9 studies met the criteria described above for meta-analysis. The search and screening processes are shown in Fig. 1. As summarized in Table 1, the 9 studies examined PEDF protein expression in cancer tissues by immunostaining semi-quantitatively. With a case–control design, they compared the clinical features, such as age, gender, tumor TNM stage and pathological grade, between PEDF high-expression group and low-PEDF expression group. Although we reviewed literatures on 14 types of primary solid tumor malignancies, the selected studies reported PEDF protein expression in 8 cancer types. The quality of the selected studies was evaluated by NOS manual as shown in Table S2. All the studies were identified as low risk for bias (NOS score ≥ 7).

**Table 1:** Summary of Studies Included in Meta-Analysis

Investigators		Zhou [40]	Zhang [42]		Li [43]		Hou [37]		Yi [38]		Uehara [41]		Jang [55]		Lv [56]		Jiang [39]	
Publication year		2016	2006		2019		2017		2016		2004		2012		2016		2010	
Cancer type		IDC	NSCLC		HCC		HCC		CRC		PDAC		TCC		PTC		RCC	
PEDF detection		IHC	IHC		IHC		IHC		IHC		IHC		IHC		IHC		IHC	
Sample size		119	91		68		149		197		80		99		271		203	
Number of patients with High-PEDF expression in each category below (%)																		
Age																		
Younger		26 (42.6%)	22 (51.2%)		25 (78.1%)		75 (62%)		79 (55.6%)		14 (29.8%)		n/a		40 (26%)		72 (55%)	
Older		26 (44.8%)	21 (43.8%)		28 (77.8%)		19 (70.4%)		31 (56.4%)		8 (24.2%)		n/a		31 (26.5%)		44 (61.1%)	
Cut-off (year old)		50	60		50		60		65		65		n/a		45		60	
Gender																		
Male		n/a	28 (44.4%)		45 (76.3%)		81 (66.4%)		70 (61.4%)		11 (24.4%)		n/a		7 (29.2%)		70 (55.6%)	
Female		52 (43.7%)	15 (53.6%)		8 (88.9%)		14 (51.9%)		40 (48.2%)		11 (31.4%)		n/a		64 (25.9%)		46 (59.7%)	
Histology Grade																		
Low Grade		3 (23.1%)	37 (46.3%)		35 (83.3%)		50 (70.4%)		84 (60.9%)		9 (28.1%)		6 (13.6%)		n/a		68 (67.3%)	
High Grade		49 (46.2%)	6 (54.5%)		15 (65.2%)		45 (57.7%)		26 (44.1%)		13 (27.1%)		7 (12.7%)		n/a		48 (47.1%)	
Cut-off		G1 vs. G2-3	W-M vs. P^*^		W-M vs. P^*^		G1-2 vs. G3-4		G1-2 vs. G3-4		G1 vs. G2-3		Low vs. High		n/a		G1-2 vs. G3-4	
TNM staging																		
Low Stage		35 (46.1%)	33 (55%)		31 (88.6%)		n/a		61 (60.4%)		8 (47.1%)		n/a		69 (28.2%)		87 (65.4%)	
High Stage		17 (39.5%)	10 (32.3%)		22 (66.7%)		n/a		39 (45.3%)		14 (22.2%)		n/a		2 (7.7%)		29 (41.4%)	
Cut-off		I–II vs. III–IV	I–II vs. III–IV		I + II vs. III		n/a		I–II vs. III		II vs III + IV		n/a		T1 vs T3		pT1-2 vs.pT3-4	
Tumor Size																		
Small Tumor		26 (55.3%)	33 (47.8%)		n/a		47 (65.3%)		43 (62.3%)		16 (32%)		n/a		48 (30.8%)		59 (60.8%)	
Large Tumor		26 (36.1%)	10 (45.5%)		n/a		42 (59.2%)		67 (52.3%)		6 (20%)		n/a		23 (20%)		67 (63.2%)	
Cut-off		2 cm	T1 vs T2-4		n/a		5 cm		T0-2 vs. T3-4		3.5 cm		n/a		1 cm		4 cm	
LN invasion																		
Negative		22 (40.7%)	27 (55.1%)		n/a		91 (62.8%)		60 (60.6%)		8 (34.8%)		n/a		59 (31.9%)		86 (58.9%)	
Positive		30 (46.2%)	16 (38.1%)		n/a		3 (100%)		32 (40%)		14 (24.6%)		n/a		12 (14%)		30 (52.6%)	
Distal metastasis																		
Negative		n/a	n/a		n/a		n/a		n/a		14 (35.9%)		n/a		n/a		97 (57.1%)	
Positive		n/a	n/a		n/a		n/a		n/a		3 (12.5%)		n/a		n/a		19 (57.6%)	
Kaplan–Meier curve																		
PEDF level in the longer OS group		High	High		Uncertain		Low		High		High		n/a		n/a		High	
Significance		P < 0.05	P < 0.05		P > 0.05		P < 0.05		P < 0.05		P < 0.05		n/a		n/a		P < 0.05	

*TCC* Transitional cell carcinoma, *IDC* intra-ductal carcinoma, *CRC* colorectal carcinoma, *PDAC* pancreatic ductal adenocarcinoma, *HCC* hepatocellular carcinoma, *NSCLC* non-small cell lung cancer, *RCC* renal cell carcinoma, *PTC* papillary thyroid carcinoma, *IHC* immunohistochemistry, *W* well-differentiated, *M* moderately-differentiated, *P* poorly-differentiated, *n/a* not available, *pT* primary tumor, *LN* lymph node, *OS* overall survival

“*” well-differentiated and moderately-differentiated groups from the original study were combined as “low grade” while poorly-differentiated group was categorized as “high grade”

### Association of PEDF protein expression and TNM staging

**Fig. 2:**
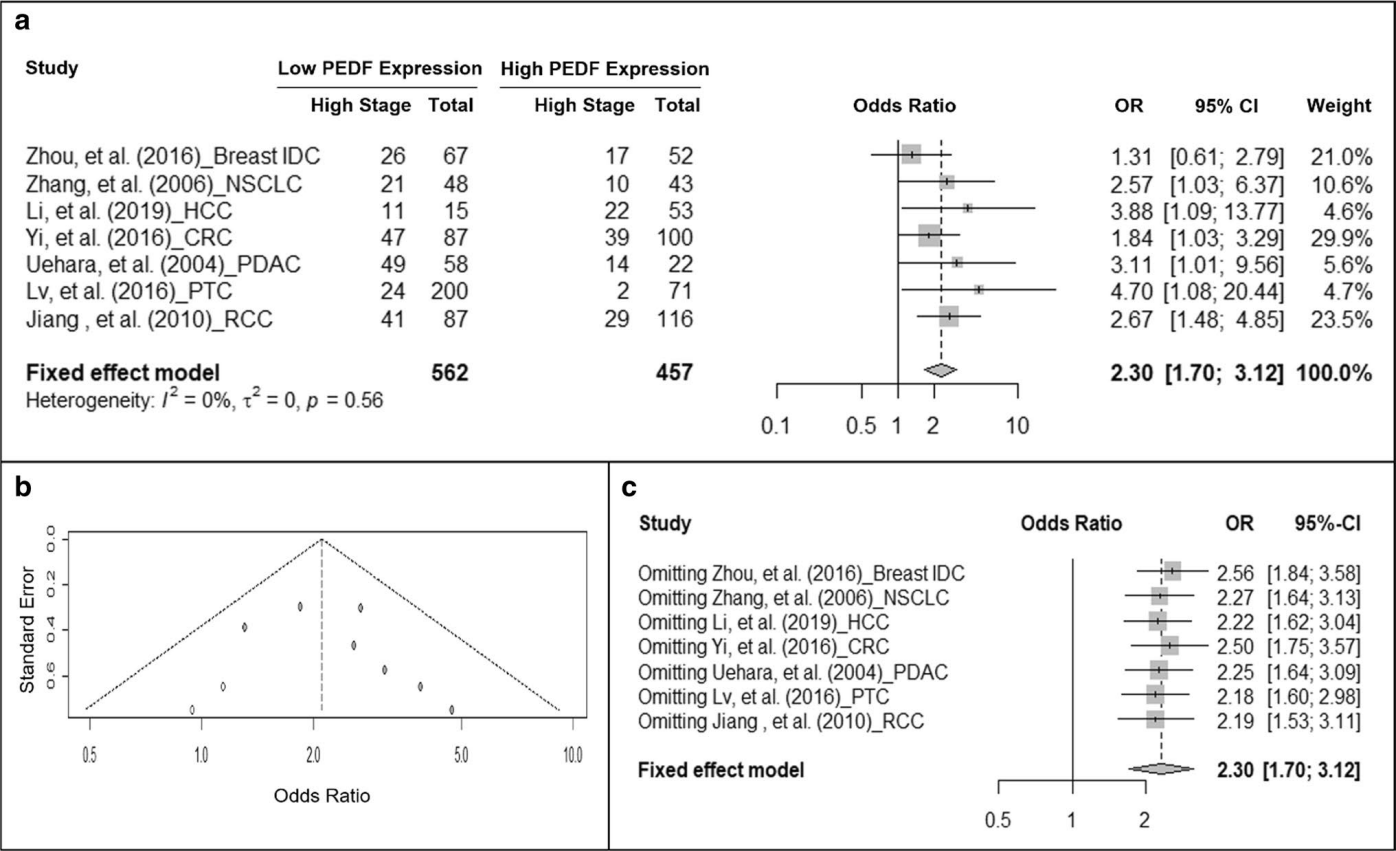
Cancer with low PEDF expression has higher odds to have higher TNM staging. **a** Forest plot showing the association of PEDF expression and cancer TNM staging. Squares indicate study-specific odds ratios (ORs). The size of the box is proportional to the weight of the study. Horizontal lines indicate 95% confidence intervals (CI). A diamond indicates the summary OR with its corresponding 95% CI. **b** Funnel plot on the publication bias of studies on PEDF expression and TNM staging. Each filled circle represents one study. Open circles represent added artificial studies to correct publication bias. **c** Forest plot of sensitivity test. Squares indicate the summary OR after omitting one specific study. Horizontal lines indicate 95% CI. A diamond indicates the summary OR of all the studies with its corresponding 95% CI. *IDC* intra-ductal carcinoma, *NSCLC* non-small cell lung cancer, *HCC* hepatocellular carcinoma, *CRC* colorectal carcinoma, *PDAC* pancreatic ductal adenocarcinoma, *PTC* papillary thyroid carcinoma, *RCC* renal cell carcinoma

The first analysis we performed is to examine if PEDF protein expression is associated with cancer progression clinically manifested by TNM staging. TNM stage II and below were classified as “low stage” while stage III and above were classified as “high stage”. The pooled effect from those studies were shown in Fig. 2a. Low PEDF-expressing tumors had increased odds to be high TNM stage compared to high PEDF-expressing tumors (OR = 2.30, 95% CI: 1.70–3.12, fixed-effect model, *I*^2^ = 0%, P = 0.56). Publication bias was found and 2 artificial studies were added by the “meta” package to correct the pooled OR (Fig. 2b). The adjusted odds ratio remained significant (OR = 2.13, 95% CI: 1.61–2.81, *I*^2^ = 0%, P = 0.53). Sensitivity analysis (Fig. 2c) suggested that none of the individual study significantly affected the pooled effect.

### Association of PEDF protein expression and tumor size

**Fig. 3:**
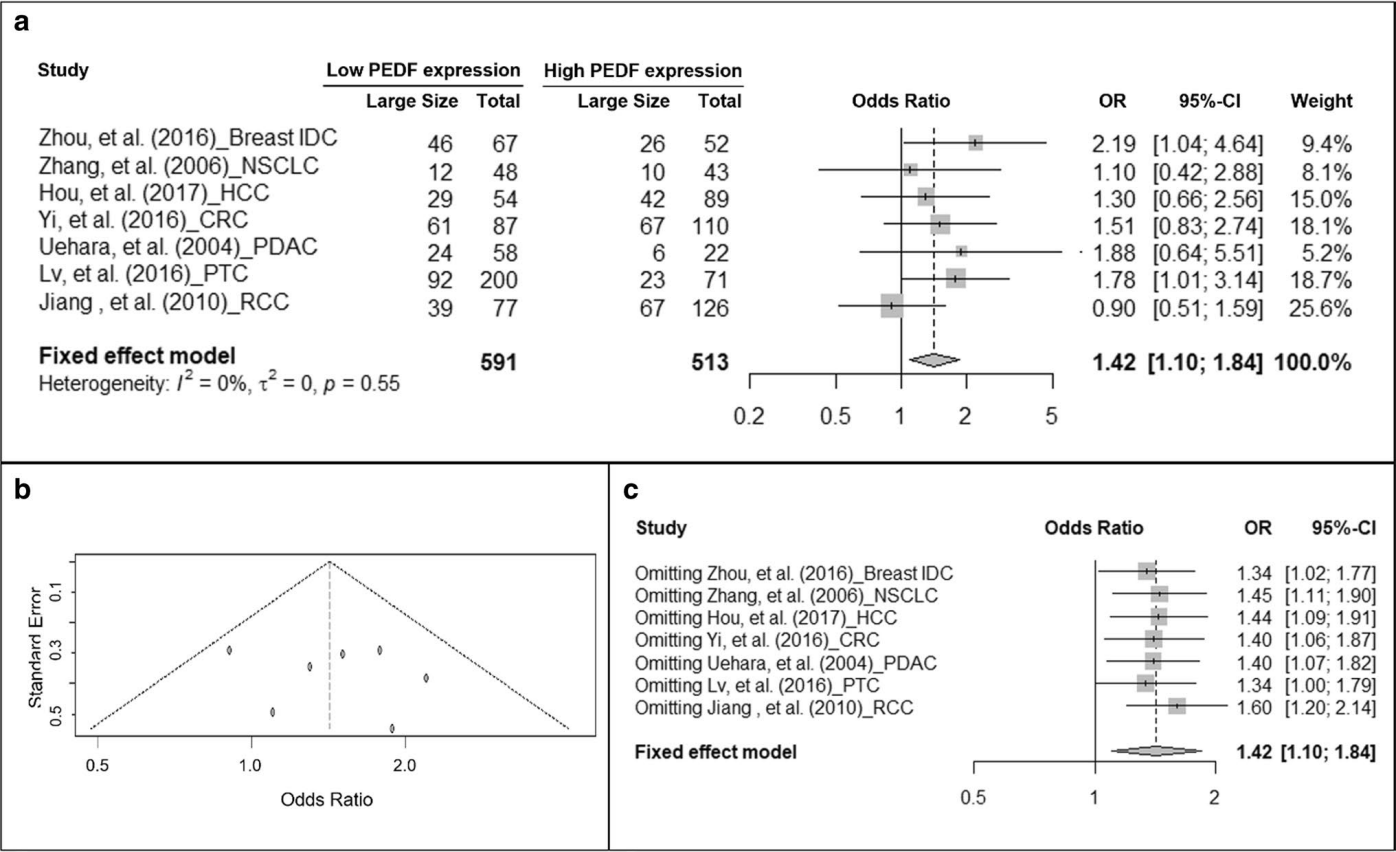
Cancer with low PEDF protein expression is larger in size. **a** Forest plot showing the association of PEDF expression and tumor size. Squares indicate study-specific odds ratios (ORs). The size of the box is proportional to the weight of the study. Horizontal lines indicate 95% confidence interval (CI). A diamond indicates the summary OR with its corresponding 95% CI. **b** Funnel plot showing no publication bias of studies on PEDF expression and tumor size. Each filled circle represents one study. **c** Forest plot of sensitivity test. Squares indicate the summary OR after omitting one specific study. Horizontal lines indicate 95% CI. A diamond indicates the summary OR of all the studies with its corresponding 95% CI. *IDC* intra-ductal carcinoma, *NSCLC* non-small cell lung cancer, *HCC* hepatocellular carcinoma, *CRC* colorectal carcinoma, *PDAC* pancreatic ductal adenocarcinoma, *PTC* papillary thyroid carcinoma, *RCC* renal cell carcinoma

We further investigated if PEDF protein expression is correlated with tumor size, lymph node invasion, and distal metastasis, each of which could contribute to tumor progression. Although different studies adopted different cut-off values to classify “small-” and “large-” tumors as shown in Table 1, each threshold is consistent with the feature of a specific type of cancer and is conventionally used to classify tumors of that type based on size. Therefore, we did not use any absolute value to define tumor size; instead, we directly extracted and pooled data based on the classification in each original study. Meta-analysis showed that low PEDF expression is associated with larger tumor size compared to high PEDF expression, presenting an increased odds (OR = 1.42, 95% CI: 1.1–1.84, fixed-effect model, *I*^2^ = 0%, P = 0.55) (Fig. 3a). No publication bias was detected (Fig. 3b). In the sensitivity test, omitting any studies did not change the conclusion (Fig. 3c).

### Association of PEDF protein expression with local or distal invasion

**Fig. 4:**
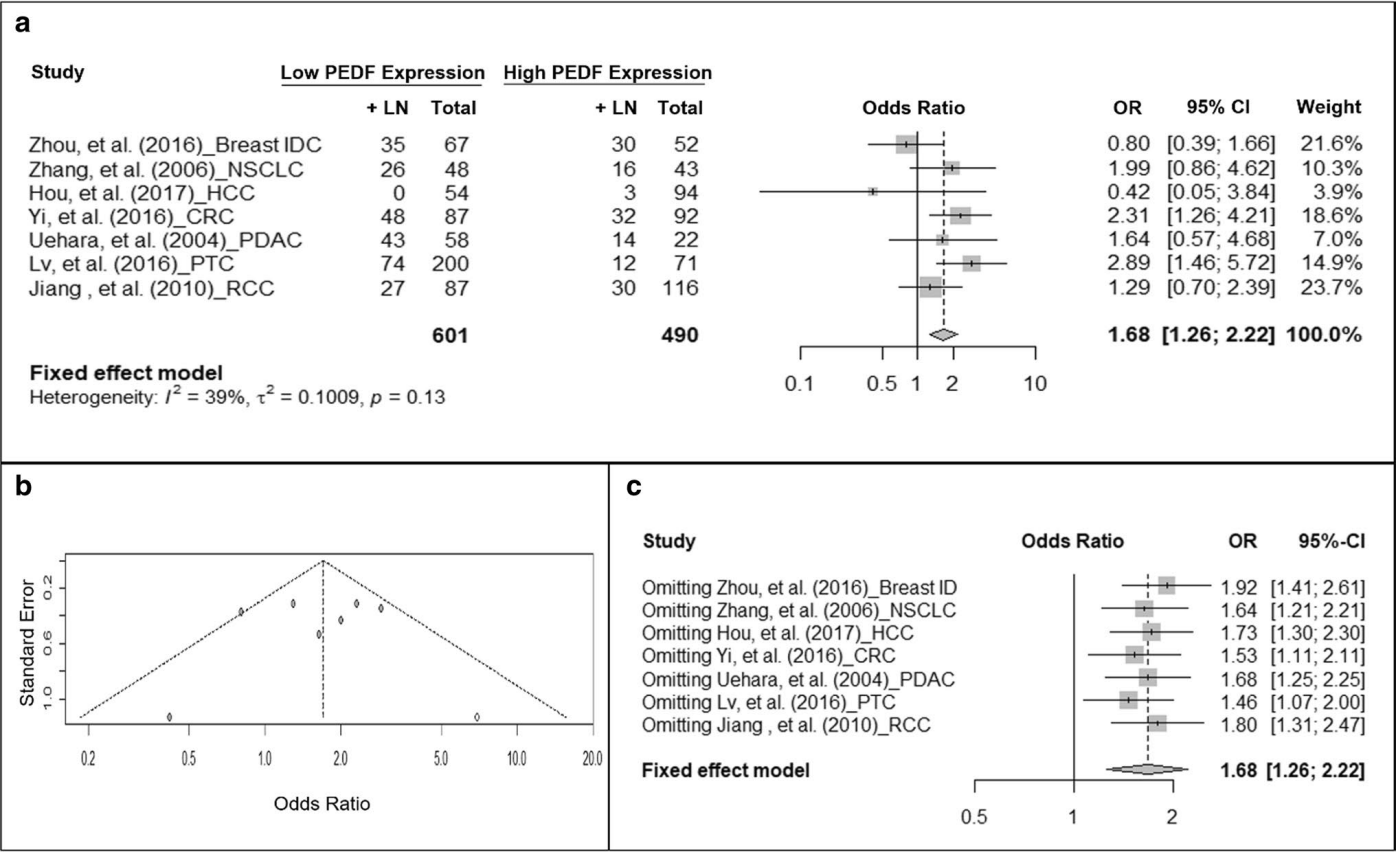
Cancer with low PEDF protein expression has higher odds to invade lymph nodes. **a** Forest plot showing the association of PEDF expression and lymph node invasion. Squares indicate study-specific odds ratios (ORs). The size of the box is proportional to the weight of the study. Horizontal lines indicate 95% confidence interval (CI). A diamond indicates the summary OR with its corresponding 95% CI. **b** Funnel plot on the publication bias of studies on PEDF expression and lymph node invasion. Each filled circle represents one study. Open circles represent filled studies to correct publication bias. **c** Forest plot of sensitivity test. Squares indicate the summary OR after omitting one specific study. Horizontal lines indicate 95% CI. A diamond indicates the summary OR of all the studies with its corresponding 95% CI. *+LN* positive lymph node, *IDC* intra-ductal carcinoma, *NSCLC* non-small cell lung cancer, *HCC* hepatocellular carcinoma, *CRC* colorectal carcinoma, *PDAC* pancreatic ductal adenocarcinoma, *PTC* papillary thyroid carcinoma, *RCC* renal cell carcinoma

Next we examined if PEDF protein expression is also associated with lymph node invasion. Low PEDF-expressing tumors showed an increased odds to metastasize to lymph node compared to high PEDF-expressing tumors (OR = 1.68, 95% CI: 1.26–2.22, fixed-effect model, *I*^2^ = 39%, P = 0.13) (Fig. 4a). Publication bias was detected and one artificial study was added (Fig. 4b). After correcting the bias, the OR remained significant (OR = 1.7, 95% CI:1.28–2.27; *I*^2^ = 38%, P = 0.12). Omitting any study in the sensitivity test did not change the result (Fig. 4c). Only two studies reported the observation on PEDF expression and distal metastasis. Meta-analysis on these studies did not find a significant association between PEDF expression and distal metastasis (OR = 1.42, 95% CI: 0.75–2.67; *I*^2^ = 67%, P = 0.08) (Fig. S1).

### Association of PEDF protein expression and histological grade of cancer

**Fig. 5:**
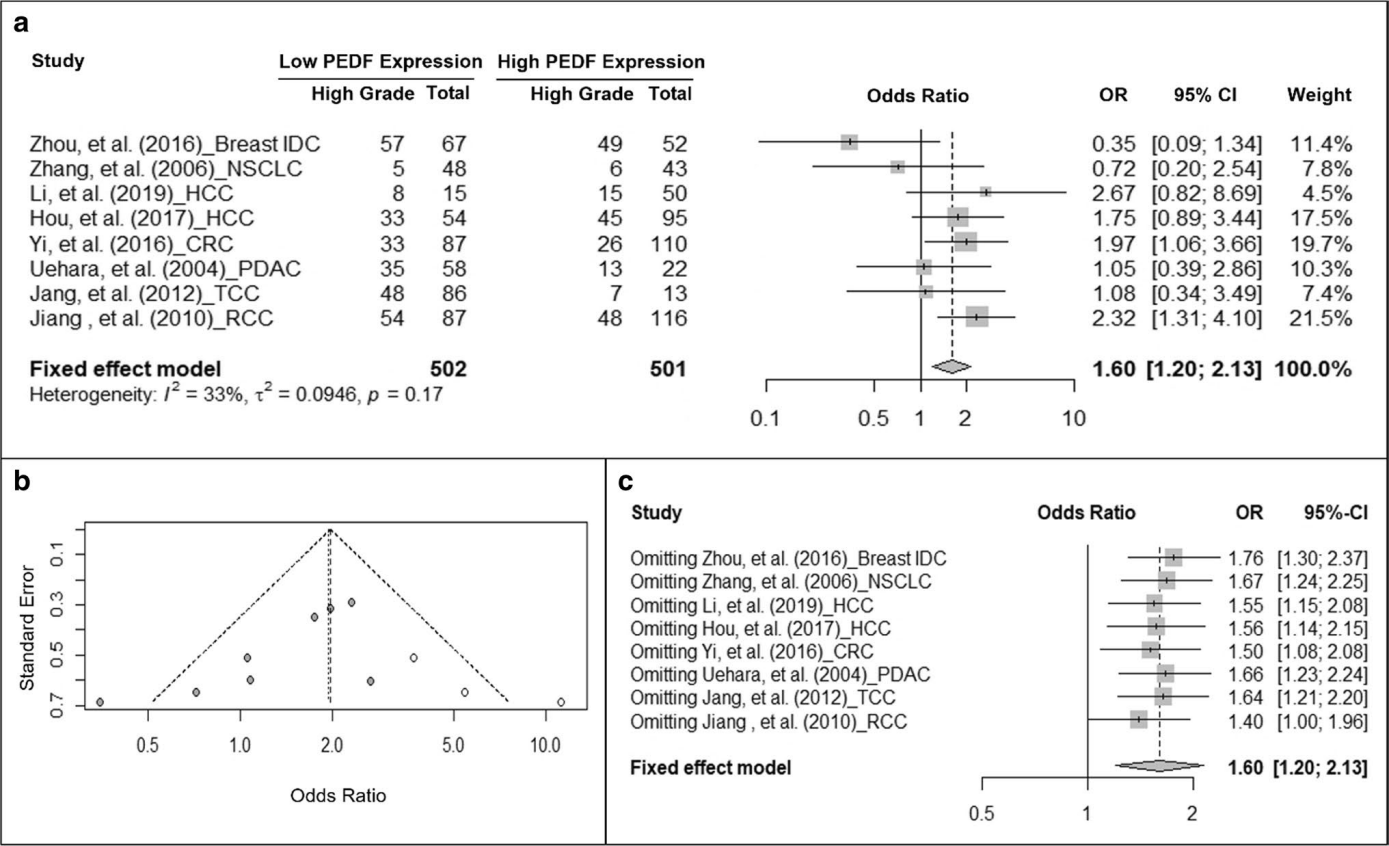
Cancer with low PEDF expression has increased odds to have higher pathological grade. **a** Forest plot showing the association of PEDF expression and tumor pathological grade. Squares indicate study-specific odds ratios (ORs). The size of the box is proportional to the weight of the study. Horizontal lines indicate 95% confidence interval (CI). A diamond indicates the summary OR with its corresponding 95% CI. **b** Funnel plot on the publication bias of studies on PEDF expression and tumor pathological grade. Each filled circle represents one study. Open circles represent filled studies to correct publication bias. **c** Forest plot of sensitivity test. Squares indicate the summary OR after omitting one specific study. Horizontal lines indicate 95% CI. A diamond indicates the summary OR of all the studies with its corresponding 95% CI. *IDC* intra-ductal carcinoma, *NSCLC* non-small cell lung cancer, *HCC* hepatocellular carcinoma, *CRC* colorectal carcinoma, *PDAC* pancreatic ductal adenocarcinoma, *TCC* (bladder) transitional cell carcinoma, *RCC* renal cell carcinoma

Other than TNM stating, we are also interested in the association of PEDF protein expression with tumor histological grades. While reviewing the original studies, we noticed that tumors had been categorized into “low-grade” and “high-grade” groups based on the conventional cut-off criteria of each type of cancer (Table 1). Specifically, Hou et al. [37], Yi et al*.* [38], and Jiang et al. [39] grouped tumors with grade G1-2 to be “low grade” and tumors with grade G3-4 to be “high grade”. Zhou et al. [40] and Uehara et al. [41] defined G1 tumors as “low grade” and G2-3 tumors as “high grade”; while Jang et al. [39] did not provide grading information for the “low grade” and “high grade” groups in their study. Zhang et al. [42] and Li et al. [43] stratified tumor grades into “well-”, “moderately-” and “poorly-differentiated”. According to the categorization by the original studies, we extracted and pooled the data into the “low-grade” and “high-grade” groups. We also combined well-differentiated and moderately-differentiated tumors in Zhang et al. [42] and Li et al. [43]’s studies into the “low-grade” group while pooled the poorly-differentiated cases into the “high-grade” group. Meta-analysis with the pooled data revealed that cancers with low PEDF expression have an increased odds to be high grade than those with high PEDF expression (OR = 1.6, 95% CI: 1.2–2.13, fixed-effect model, *I*^2^ = 33%, P = 0.17) (Fig. 5a). Funnel plot (Fig. 5b) showed the publication bias and 3 studies were added to correct the publication bias. The adjusted meta-analysis still suggested an increased odds for low PEDF-expressing tumor to be high grade (OR = 1.94, 95% CI: 1.26–2.99, randomized model, *I*^2^ = 55%, P = 0.01). Excluding any one of the studies did not change the pooled OR in sensitivity test (Fig. 5c).

### Prognostic value of PEDF

**Fig. 6:**
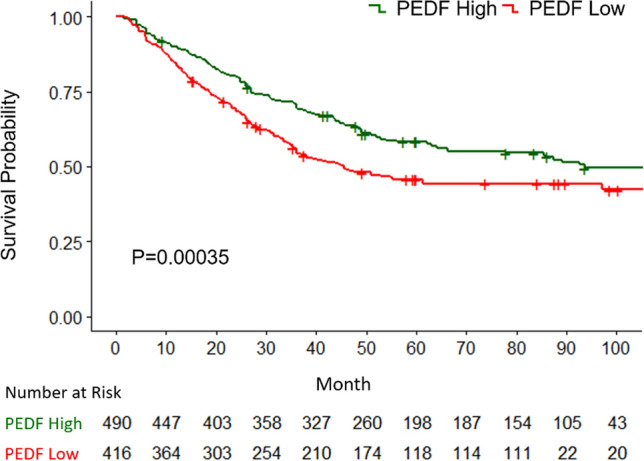
Cancer patients with high-PEDF protein expression have longer overall survival shown in Kaplan–Meier curves. Green line represents the OS of patients with high PEDF-expressing cancers whereas red line represents the OS of patients with low PEDF-expressing cancers. Below the graph shows the number of patients at risk for each group at a particular time point

Lastly, we investigated the correlation between PEDF protein expression level and the prognosis of cancer patients. As the conclusions from previous studies are not consistent (Table 1), we aim to pool the patients’ data from the original studies and to analyze the effect of PEDF protein expression on the overall survival of cancer patients. We used a novel method to reconstruct individual patient data from the published Kaplan–Meier curve following the protocol developed by Guyot, et al. [33]. After combining the data of a total of 906 individual patients, we plotted a Kaplan–Meier curve to compare the overall survival of patients with high PEDF protein expression and that of patients with low PEDF protein expression. Patients with high-PEDF protein expression in the cancer tissue have a significant longer overall survival than those with low-PEDF protein expression (P = 0.00035) (Fig. 6).

We also studied if PEDF mRNA level is correlated with patients’ survival on a pan-cancer basis with the data from The Cancer Genomic Atlas (TCGA), a cancer genomic program established by the National Cancer Institute (https://www.cancer.gov/tcga). We used Oncolnc, an online platform that incorporates TCGA database and provides gene expression-survival data [34]. A total of 7970 individual patient data of 21 malignancies were downloaded and pooled. The analysis of the correlation between PEDF mRNA and overall survival was performed. To our surprise, low PEDF expression at mRNA level is associated with longer survival time (with a marginal statistical significance P = 0.045, Fig. S2). The finding is the opposite of the observation on the PEDF protein-survival correlation (Fig. 6). The discrepancy could likely be explained by the fact that gene expression at the mRNA level and gene expression at the protein level are not consistent in many scenarios [44]. Many layers of regulation such as translation efficiency, post-translational modification and protein degradation have impact on protein concentration independent of the change of mRNA quantity [44, 45]. In addition, mRNA level is much more susceptible to fluctuation than protein upon the transition of cell status. It was found that the global mRNA number in a cell shrinks substantially when the cell switches from a proliferating state to quiescence while the global protein number only drops by ~ 9.5% [46]. Therefore, we make the following hypothesis to explain the contradictory data: the aggressive tumors associated with shorter overall survival likely have more proliferating cells and thus higher global mRNA level including PEDF mRNA; meanwhile, PEDF protein expression in those tumors is largely suppressed due to the translation and/or post-translation regulation. On the other hand, tumors associated with longer survival have relatively more cells in quiescence and significantly lower global mRNA including PEDF mRNA; however, PEDF protein translation is very efficient in those tumors which results in higher PEDF protein expression.

### Association of PEDF protein expression and age or gender

According to the NOS manual, it is not known if patients with different gender or age are evenly distributed between high PEDF-expression group and low PEDF-expression group. Thus, gender or age could be confounding factors in the analysis of the association between PEDF expression and cancer progression or overall survival. For example, if the average age in low-PEDF expression group is higher, this group would likely show a shorter overall survival compared to high-PEDF expression group. To assess this possibility, we performed meta-analysis on the association of PEDF expression with gender or age. PEDF expression was neither associated with the patients’ gender (OR = 0.91, 95% CI: 0.68–1.22, fixed-effect model, *I*^2^ = 16%, P = 0.3) (Fig. S3), nor associated with their age (using 50 years old as cut-off, pooled effect of three studies: OR = 0.96, 95% CI: 0.64–1.44, fixed-effect model, *I*^2^ = 0%, P = 0.98, Fig. S4a; using 60 years old as cut-off, pooled effect of five studies: OR = 0.94, 95% CI: 0.68–1.3, fixed-effect model, *I*^2^ = 0%, P = 0.73, Fig. S4b). The results suggested that gender and age are not confounding factors.

## Discussion

This is the first systemic review and meta-analysis of PEDF expression in major types of human cancer since the identification of PEDF three decades ago. This study shows that the decreased PEDF expression at protein level is associated with more adverse clinical outcomes in cancer patients, manifested by higher TNM staging, higher tumor grading, and a significantly shorter overall survival. Using meta-analysis, this study provides a quantified strength of the association between PEDF and the clinical features of malignancy in a pan-cancer manner. Thus this study reveals the value of PEDF as a general biomarker to assess cancer progression and prognosis in cancer management. In addition, the result of this study is consistent with the findings in preclinical models that PEDF has multifaceted anti-tumor activities and suggests the broad anti-tumor effect of PEDF in a wide range of human malignancies.

Despite the anti-angiogenesis and anti-tumor cell activity of PEDF, we did not find the association between PEDF expression and distal metastasis. This could be due to the lack of detection power as only two studies were available for meta-analysis. Biologically, the local PEDF expression in the primary tumor may not influence the tumor implantation at a distal tissue/organ, where a tumor-favorable microenvironment is necessary for successful metastasis [47]. In fact, it would be interesting to study if systemic PEDF protein level, such as PEDF serum concentration, is correlated with local invasion of primary tumors and distal metastasis.

The relation of PEDF mRNA-survivorship does not agree with the finding that low PEDF expression at protein level is correlated with poorer overall survival. Although the central dogma of biology described the tight link from DNA to RNA and to protein, transcript level by themselves are not sufficient to predict protein levels and to explain genotype–phenotype relationships in many scenarios [44]. Previous research found that the levels of mRNAs and their corresponding proteins showed limited correlation (*R*^2^ = 0.55 in cycling cells) globally [46, 48] and only 40% of the variance of protein levels could be explained by mRNA levels [49]. Besides, protein level is more stable relative to mRNA level under unsteady state. As protein is the molecule that realizes the gene function and shows higher stability in quantity, the prognostic value of protein is probably higher than that of transcripts. To gain more knowledge on how PEDF expression correlates with clinical prognosis, more studies on PEDF at both transcriptomic and proteomic levels are needed.

Other than the predictive value of PEDF protein in caner progression and prognosis, measuring PEDF protein expression in cancer tissue might guide cancer treatment, especially the usage of anti-angiogenic therapy. The anti-angiogenic therapies approved by Food and Drug Administration (FDA) are primarily targeting the vascular endothelial growth factor (VEGF) pathway, such as the monoclonal antibody against VEGF [50] or chemical inhibitors of tyrosine kinase receptors stimulated by VEGF [51, 52]. However, the use of anti-angiogenic therapy is not individualized to minimize the therapy-related adverse events. Like endocrine therapy, which is indicated specifically by the expression of hormonal receptors in cancer tissue [53, 54], it would be interesting to know if any pathological feature could specifically suggest the sensitivity of tumors to anti-angiogenic reagents. Given that PEDF is the strongest endogenous anti-angiogenic factor that counteracts multiple pro-angiogenic factors including VEGF, the down-regulation of PEDF in cancer tissue could suggests a tilted balance toward over-angiogenic. Therefore, it is worth researching if PEDF expression in cancer tissue could be a useful indication for anti-angiogenic therapy. In addition, the inverse correlation between PEDF expression and tumor progression in multiple human tumor types also suggests the potential clinical value of developing cancer treatment by targeting this pathway.

In conclusion, we reviewed studies on PEDF expression in 14 solid tumor malignancies with the highest incidence worldwide. Nine case–control studies on eight types of cancers were selected for meta-analysis. The pooled result suggested that low PEDF protein expression in cancer tissue is significantly associated with cancer progression. Low PEDF expression in cancer tissue is also correlated with a poorer survival. This study indicates a potential clinical value of PEDF as a prognostic marker and as an indicator for anti-angiogenesis therapy.

## Supplementary Information

**Additional file 1: Table S1**. Summary of Search Strategy. **Table S2**. Quality Assessment of Selected Studies by Newcastle-Ottawa Scale (NOS)

**Additional file 2: Fig S1**. PEDF expression in cancer tissue is not associated with distal metastasis. Forest plot showing the association of PEDF expression and distal metastasis. Squares indicate study-specific odds ratios (ORs). The size of the box is proportional to the weight of the study. Horizontal lines indicate 95% confidence interval (CI). A diamond indicates the summary OR with its corresponding 95% CI. +Mets: positive for distal metastasis, PDAC: pancreatic ductal adenocarcinoma, RCC: Renal cell carcinoma. **Fig. S2**. Correlation of PEDF mRNA and overall survival. Low PEDF mRNA level in cancer is associated with longer survival probability with marginal statistical significance. **Fig. S3**. PEDF expression in cancer tissue is not associated with the gender of patients. Forest plot showing the association of PEDF expression and the gender of patients. Squares indicate study-specific odds ratios (ORs). The size of the box is proportional to the weight of the study. Horizontal lines indicate 95% confidence interval (CI). A diamond indicates the summary OR with its corresponding 95% CI. NSCLC: non-small cell lung cancer, HCC: hepatocellular carcinoma, CRC: colorectal carcinoma, PDAC: pancreatic ductal adenocarcinoma, PTC: papillary thyroid carcinoma, RCC: Renal cell carcinoma. **Fig. S4**. PEDF expression in cancer tissue is not associated with the age of patients. (a) Forest plot showing the association of PEDF expression and patients’ age (50 years old as cut-off). Squares indicate study-specific odds ratios (ORs). The size of the box is proportional to the weight of the study. Horizontal lines indicate 95% confidence interval (CI). A diamond indicates the summary OR with its corresponding 95% CI. (b). Forest plot showing the association of PEDF expression and patients’ age (60 years old as cut-off). Squares indicate study-specific odds ratios (ORs). The size of the box is proportional to the weight of the study. Horizontal lines indicate 95% confidence interval (CI). A diamond indicates the summary OR with its corresponding 95% CI. NSCLC: non-small cell lung cancer, HCC: hepatocellular carcinoma, CRC: colorectal carcinoma, PDAC: pancreatic ductal adenocarcinoma, PTC: papillary thyroid carcinoma, RCC: renal cell carcinoma.

## Data Availability

The datasets generated during and/or analyzed during the current study are available from the corresponding author on reasonable request.

## Abbreviations

AMD: Age-related macular degeneration
CI: Confidence interval
CRC: Colorectal carcinoma
DR: Diabetic retinopathy
FDA: Food and Drug Administration
HCC: Hepatocellular carcinoma
IDC: (Breast) intra-ductal carcinoma
IHC: Immunohistochemistry
IPD: Individual patient data
KMC: Kaplan–Meier curve
LN: Lymph node
n/a: Not available
NOS: Newcastle–Ottawa Scale
NSCLC: Non-small cell lung cancer
OR: Odds ratio
OS: Overall survival
PDAC: Pancreatic ductal adenocarcinoma
PEDF: Pigment epithelial derived factor
PLXDC1: Plexin-domain containing protein 1
PLXDC2: Plexin-domain containing protein 2
pT: Primary tumor
PTC: Papillary thyroid carcinoma
RCC: Renal cell carcinoma
SERPINF1: Serine protease inhibitor F1
TCC: Transitional cell carcinoma;
TEM7: Tumor endothelial marker 7
VEGF: Vascular endothelial growth factor

## Code availability

The code used in the current study are available from the corresponding author on reasonable request.
