# Supplementary material for: Association of pigment epithelium derived factor expression with cancer progression and prognosis: a meta-analysis study
Source: Discov Oncol. 2021 Dec 15;12:61. doi: 10.1007/s12672-021-00457-y (PMC8777498; doi:10.1007/s12672-021-00457-y)
Supplement: Supplementary file 1 — Additional file 1: Table S1. Summary of Search Strategy. Table S2. Quality Assessment of Selected Studies by Newcastle-Ottawa Scale (NOS) [file 12672_2021_457_MOESM1_ESM.docx]

**Supplemental Table 1.** Summary of Search Strategy

| General search strategy | PubMed: PEDF entry” AND (XX cancer[MeSH Terms] OR (XX organ AND Malignancy entry))  EMBASE: “PEDF entry AND (XX cancer [Emtree] OR (XX organ AND Malignancy entry)) | |
| --- | --- | --- |
| Search entry | Search built in PubMed | Search built in EMBASE |
| PEDF entry | "PEDF" OR "pigment epithelium derived factor" OR "SERPINF1" OR "serine proteinase inhibitor F1" [Baumeister 2019] | pigment epithelium derived factor'/exp OR 'serpinf1 protein'/exp OR 'serpinf1 gene'/exp OR 'pedf gene'/exp OR 'pigment epithelium derived factor':ab,ti OR 'pedf':ab,ti OR 'serine protease inhibitor f1':ab,ti OR 'serpinf1':ab,ti |
| Malignancy entry | malign*[Title/Abstract] OR "neoplasm*"[Title/Abstract] OR "carcinoma*"[Title/Abstract] OR "cancer*"[Title/Abstract] OR "tumor*"[Title/Abstract] OR "tumour"[Title/Abstract] | malign*:ti,ab OR neoplasm*:ti,ab OR carcinoma*:ti,ab OR cancer*:ti,ab OR tumor*:ti,ab OR tumour*:ti,ab OR angiosarcoma:ti,ab |
| Breast cancer  (WHO ranking^*^:1) | breast neoplasms[MeSH Terms] OR (("breast"[Title/Abstract] OR "mammary gland"[Title/Abstract]) AND "Malignancy entry") | ('breast tumor'/exp OR ((breast:ab,ti OR 'mammary gland':ab,ti) AND Malignancy entry) |
| Lung cancer  (WHO ranking^*^:2) | "lung neoplasms"[MeSH Terms] OR ("lung"[Title/Abstract] AND "Malignancy entry") | ('lung cancer'/exp OR 'lung tumor'/exp OR ((lung:ab,ti) AND Malignancy entry) |
| Colorectum cancer  (WHO ranking^*^:3) | "colorectal neoplasms"[MeSH Terms] OR (("colon"[All Fields] OR "colonic"[All Fields] OR "colonal"[All Fields] OR "rectum"[All Fields] OR "rectal"[All Fields] OR "colorectal"[All Fields] OR "colorectum"[All Fields]) AND "Malignancy entry") | ('colorectal cancer'/exp OR 'colorectal tumor'/exp OR 'colon cancer'/exp OR 'rectum cancer'/exp OR 'rectum tumor'/exp OR 'colon tumor'/exp) OR ((colon:ab,ti OR 'rectum':ab,ti OR 'rectal':ab,ti OR 'colorectal':ab,ti OR 'colorectum':ab,ti) AND Malignancy entry) |
| Prostate cancer  (WHO ranking^*^: 4) | "prostatic neoplasms"[MeSH Terms] OR (("prostate"[Title/Abstract] OR "prostatic [All Fields]) AND "Malignancy entry") | ('pancreas cancer'/exp OR 'pancreas tumor'/exp OR 'pancreas carcinoma'/exp) OR ((pancreas:ab,ti OR pancreatic:ab,ti OR pancreatic:ab,ti) AND Malignancy entry) |
| Liver cancer  (WHO ranking^*^: 6) | liver neoplasms[MeSH Terms] OR "biliary tract neoplasms"[MeSH Terms] OR (("liver"[Title/Abstract] OR "hepatocellular"[Title/Abstract] OR "cholangio*"[Title/Abstract] OR "hepatoblastoma"[Title/Abstract] OR "hepatic"[Title/Abstract] OR "hepatoma"[Title/Abstract] OR "hepatocarcinoma"[Title/Abstract] OR "gallbladder"[Title/Abstract] OR "biliary tract"[Title/Abstract] OR "bile ducts"[Title/Abstract]) AND "Malignancy entry") | (('liver cancer'/exp OR 'biliary tract cancer'/exp) OR (('liver':ti,ab OR 'hepatocellualr':ti,ab OR 'cholangio*':ti,ab OR 'hepatoblastoma':ti,ab OR 'hepatic':ti,ab OR 'hepatoma':ti,ab OR 'hepatocarcinoma':ti,ab OR 'gallbladder':ti,ab OR 'biliary tract':ti,ab OR 'bile ducts':ti,ab) AND Malignancy entry) |
| stomach cancer  (WHO ranking^*^: 5) | "stomach neoplasms"[MeSH Terms] OR (("stomach"[Title/Abstract] OR "gastric"[Title/Abstract] OR "gastral"[Title/Abstract]) AND "Malignancy entry") | ('stomach cancer'/exp OR ((gastric:ab,ti OR gastral:ab,ti OR 'stomach':ab,ti) AND Malignancy entry) |
| Cervical cancer  (WHO ranking^*^: 7) | "uterine cervical neoplasms"[MeSH Terms] OR ("cervical"[Title/Abstract] OR "cervix"[Title/Abstract]) AND "Malignancy entry") | uterine cervix cancer'/exp OR ((cervical:ab,ti OR cervix:ab,ti) AND Malignancy entry) |
| Oesophagus cancer  (WHO ranking^*^: 8) | "esophageal neoplasms"[MeSH Terms] OR (("esophageal"[Title/Abstract] OR "esophagus"[Title/Abstract])AND "Malignancy entry") | ('esophagus cancer'/exp OR ((esophageal:ab,ti OR esophagus:ab,ti) AND Malignancy entry) |
| Thyroid cancer  (WHO ranking^*^: 9) | "thyroid neoplasms"[MeSH Terms] OR ("thyroid"[Title/Abstract] AND "Malignancy entry") | ('thyroid cancer'/exp OR 'thyroid tumor'/exp OR 'thyroid carcinoma'/exp) OR (('thyroid':ab,ti) AND Malignancy entry) |
| Bladder cancer  (WHO ranking^*^: 10) | "urinary bladder neoplasms"[MeSH Terms] OR ("bladder"[Title/Abstract] AND "Malignancy entry") | bladder cancer'/exp OR 'bladder tumor'/exp OR (bladder:ab,ti AND Malignancy entry) |
| Non-Hodgkin lymphoma  (WHO ranking^*^: 11) | "lymphoma, non hodgkin"[MeSH Terms] OR "lymphoma"[MeSH Terms] OR "non hodgkin lymphoma"[Title/Abstract] OR "non hodgkin s lymphoma"[Title/Abstract] OR "NHL"[Title/Abstract] OR "lymphoma" | ('nonhodgkin lymphoma'/exp OR 'nonhodgkin lymphoma':ab,ti OR 'nhl':ab,ti) |
| Pancreatic cancer  (WHO ranking^*^: 12) | "pancreatic neoplasms"[MeSH Terms] OR ("pancrea*"[All Fields] AND "Malignancy entry") | ('pancreas cancer'/exp OR 'pancreas tumor'/exp OR 'pancreas carcinoma'/exp) OR ((pancreas:ab,ti OR pancreatic:ab,ti OR pancreatic:ab,ti) AND Malignancy entry) |
| Kidney cancer  (WHO ranking^*^: 14^#^) | "carcinoma, renal cell"[MeSH Terms] OR "kidney neoplasms"[MeSH Terms] OR (("kidney"[Title/Abstract] OR "renal"[Title/Abstract] OR "renal cell"[Title/Abstract]) AND "Malignancy entry") | ('kidney cancer'/exp OR 'kidney tumor'/exp OR 'renal cell carcinoma'/exp) OR (('kidney':ti,ab OR 'renal':ti,ab OR 'renal cell':ti,ab) AND Malignancy entry) |
| Uterus cancer  (WHO ranking^*^: 15) | "uterine neoplasms"[MeSH Terms] OR (("uterine"[Title/Abstract] OR "uterus"[Title/Abstract] OR "corpus uteri"[Title/Abstract]) AND "Malignancy entry") | ('uterus cancer'/exp OR (('uterus':ab,ti OR 'utertine':ab,ti OR 'corpus uteri':ab,ti) AND Malignancy entry) |

*: Rank of worldwide incidence in 2020 by World Health Organization (WHO). ^#^: The 13^th^ ranking cancer is leukemia and thus does not meet the criteria to be solid tumor to be included in the study.

**Supplemental Table 2.** Quality Assessment of Selected Studies by Newcastle-Ottawa Scale (NOS)

NOS Assessing Items

| Study | | Zhou (2016) | Zhang (2006) | Li (2019) | Hou (2017) | Yi (2016) | Uehara (2004) | Jang (2012) | Lv (2016) | Jiang (2010) |
| --- | --- | --- | --- | --- | --- | --- | --- | --- | --- | --- |
| Selection | The case definition is adequate with independent validation | * | * | * | * | * | * | * | * | * |
|  | Consecutive or obviously representative series of cases | * | * | * | * | * | * | * | * | * |
|  | Controls derive from the same community as cases | * | * | * | * | * | * | * | * | * |
|  | Controls have explicitly different outcome from cases | * | * | * | * | * | * | * | * | * |
| Compatibility | Cases and controls have comparable treatment | * | * | - | * | * | * | - | * | * |
|  | Cases and control with comparability on any other factors (age, gender) | - | - | - | - | - | - | - | - | - |
| Exposure | Ascertainment of exposure using secure records | * | * | * | * | * | * | * | * | * |
|  | Ascertainment of exposures by the same method for cases and controls | * | * | * | * | * | * | * | * | * |
|  | Ascertainment of exposure with non-response rate for both groups | * | * | * | * | * | * | * | * | * |
| Total Quality Score | | 8 | 8 | 7 | 8 | 8 | 8 | 7 | 8 | 8 |

*:Yes. “-”: not available
