# Supplementary figures and images for: Association of pigment epithelium derived factor expression with cancer progression and prognosis: a meta-analysis study
Source: Discov Oncol. 2021 Dec 15;12:61. doi: 10.1007/s12672-021-00457-y (PMC8777498; doi:10.1007/s12672-021-00457-y)

## Supplemental Figure 1

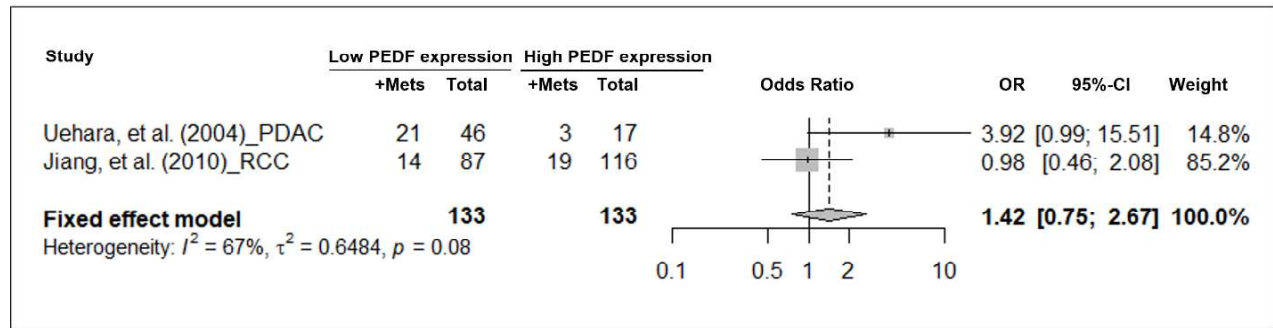

## Supplemental Figure 2

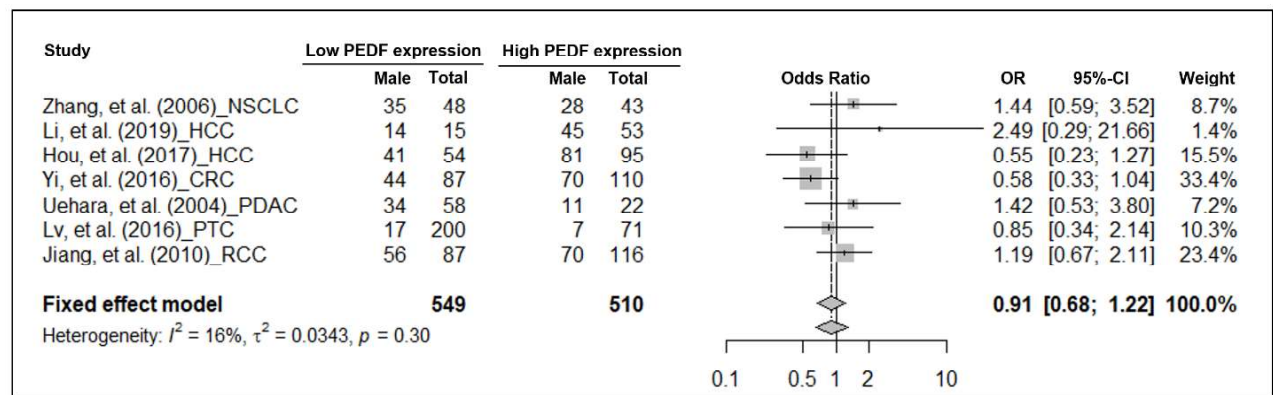

## Supplemental Figure 3

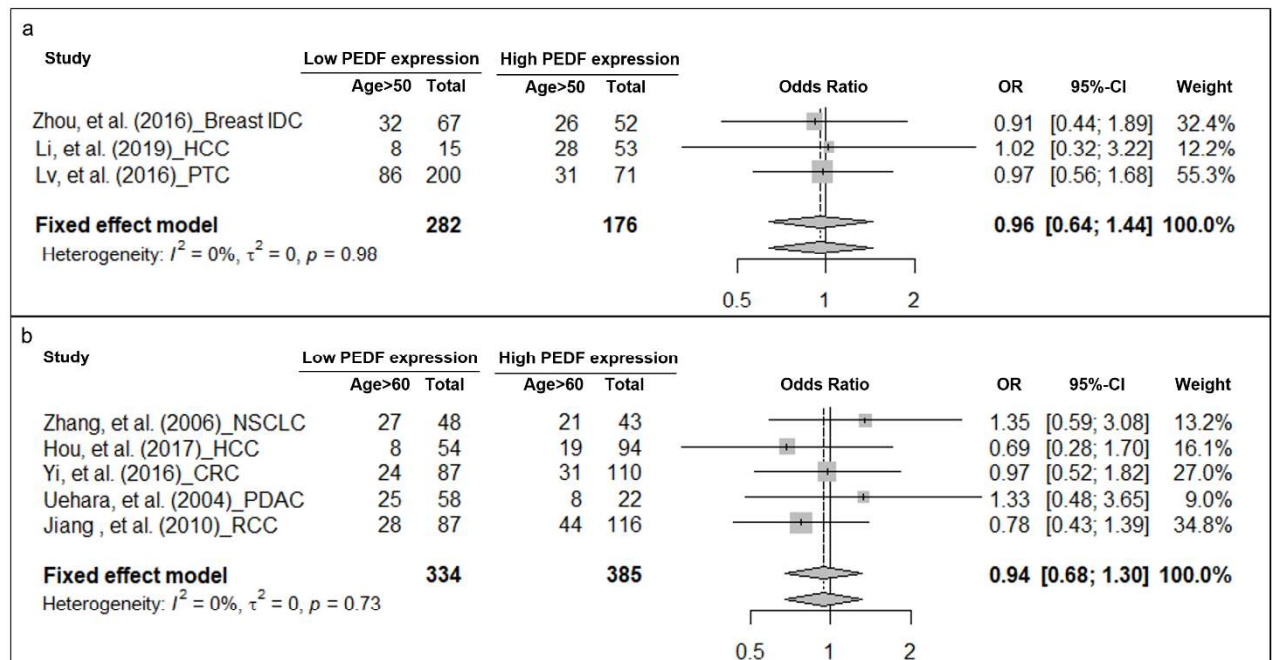

Supplement: Supplementary file 2 — Additional file 2: Fig S1. PEDF expression in cancer tissue is not associated with distal metastasis. Forest plot showing the association of PEDF expression and distal metastasis. Squares indicate study-specific odds ratios (ORs). The size of the box is proportional to the weight of the study. Horizontal lines indicate 95% confidence interval (CI). A diamond indicates the summary OR with its corresponding 95% CI. +Mets: positive for distal metastasis, PDAC: pancreatic ductal adenocarcinoma, RCC: Renal cell carcinoma. Fig. S2. Correlation of PEDF mRNA and overall survival. Low PEDF mRNA level in cancer is associated with longer survival probability with marginal statistical significance. Fig. S3. PEDF expression in cancer tissue is not associated with the gender of patients. Forest plot showing the association of PEDF expression and the gender of patients. Squares indicate study-specific odds ratios (ORs). The size of the box is proportional to the weight of the study. Horizontal lines indicate 95% confidence interval (CI). A diamond indicates the summary OR with its corresponding 95% CI. NSCLC: non-small cell lung cancer, HCC: hepatocellular carcinoma, CRC: colorectal carcinoma, PDAC: pancreatic ductal adenocarcinoma, PTC: papillary thyroid carcinoma, RCC: Renal cell carcinoma. Fig. S4. PEDF expression in cancer tissue is not associated with the age of patients. (a) Forest plot showing the association of PEDF expression and patients’ age (50 years old as cut-off). Squares indicate study-specific odds ratios (ORs). The size of the box is proportional to the weight of the study. Horizontal lines indicate 95% confidence interval (CI). A diamond indicates the summary OR with its corresponding 95% CI. (b). Forest plot showing the association of PEDF expression and patients’ age (60 years old as cut-off). Squares indicate study-specific odds ratios (ORs). The size of the box is proportional to the weight of the study. Horizontal lines indicate 95% confidence interval (CI) [file 12672_2021_457_MOESM2_ESM.pdf]
